# The Cannabis and Older Persons Study: Has Science Caught Up With Practice Yet?

**DOI:** 10.1093/geroni/igab046.1964

**Published:** 2021-12-17

**Authors:** Brian Kaskie

## Abstract

Since 2016, the Cannabis and Older Persons Study has examined the increasing use of cannabis among Americans over 60 years old. Our current work dives into particular groups of cannabis users and explores outcomes related to medical conditions and symptoms. This symposium also features a range of methodological approaches from an analysis of the BRFSS caregiving and cannabis modules, a convenience sample of more than 4,000 older cannabis users enrolled in the Illinois Medical Cannabis Program and qualitative interviews conducted with aging veterans. Kanika Arora examines the association between informal caregiving and marijuana use and whether this association varies by age. Julie Bobitt shares findings from 32 interviews with older Veteran cannabis users. Alton Croker examines cannabis use as a complement or alternative to palliative care. HyoJung Kang clusters negative outcomes experienced by older persons who use cannabis. Brian Kaskie compares cannabis use among persons with Multiple Sclerosis (N=135) and persons diagnosed with arthritis (N=582) or cancer (N=622). While we certainly find reason to remain concerned that cannabis use alone and co-occurring use with prescription opioids may contribute to increased rates of substance misuse and other undesirable outcomes among older adults, we find it increasingly difficult to overlook the benefits many persons derive when taking cannabis as a method to manage pain or address other medical conditions. At this point, public policy officials and program administrator should strive to strike a balance between addressing cannabis harms relative to promoting benefits such as opioid reduction and diversion.

